# Target Strength and swimbladder morphology of Mueller’s pearlside (*Maurolicus muelleri)*

**DOI:** 10.1038/s41598-019-53819-6

**Published:** 2019-11-21

**Authors:** B. Sobradillo, G. Boyra, U. Martinez, P. Carrera, M. Peña, X. Irigoien

**Affiliations:** 1 Azti – Marine Research, Herrera kaia, Portualdea z/g - 20110 Pasaia, (Gipuzkoa) Spain; 2 IKERBASQUE, Basque Foundation for Science, Bilbao, Spain; 3 Instituto Español de Oceanografía, Vigo, Spain; 4 Instituto Español de Oceanografía, Centro Oceanográfico de Baleares, Palma de Mallorca, Spain

**Keywords:** Ecology, Marine biology, Acoustics

## Abstract

In the last few years, there has been increasing interest in the commercial exploitation of mesopelagic fish and a trawl-acoustic methodology has been recommended to make estimates of abundance of these resources. This study provides relevant information on the scattering properties of a key mesopelagic fish species in the Bay of Biscay, Mueller’s pearlside (*Maurolicus muelleri)*, necessary to convert the acoustic density into numerical abundance. The target strength (*TS*) of pearlside was estimated for the first time at five frequencies commonly used in acoustic surveys. A high-density filter was applied to reduce the bias derived from overlapping echoes erroneously assigned to single targets. Its relationship with fish length (*b*_20_) was also determined (−65.9 ± 2, −69.2 ± 3, −69.2 ± 2, −69.5 ± 2.5 and −71.5 ± 2.5 dB at 18, 38, 70, 120 and 200 kHz, respectively). Biomass estimates of pearlside in the Bay of Biscay during the four years of study (2014–2017) are given using the 38 kHz frequency. Morphological measurements of the swimbladder were obtained from soft X-ray images and used in the backscattering simulation of a gas-filled ellipsoid. Pearlside is a physoclist species, which means that they can compensate the swimbadder volume against pressure changes. However, the best fit between the model and the experimental data showed that they lose that capacity during the trawling process, when the swimbladder volume is affected by Boyle’s law.

## Introduction

Mesopelagic fishes constitute an important component of the food web in the oceanic sound scattering layers (SSLs)^1,2^. Despite their small size, they are numerically important in temperate and tropical oceanic waters^3–5^, constituting major forage food for various commercially-fished species^6,7^. Due to the increasing interest in their commercial exploitation^8–12^, accurate estimates of its abundance are key to evaluate the impact of their exploitation and establish the necessary management measures^9,11^. Among the mesopelagic species, Mueller’s pearlside (*Maurolicus muelleri*, Gmelin, 1789; pearlside hereafter) is one of the most abundant and potentially accessible species to commercial fisheries, as it often resides close to the surface^13^.

The total abundance of mesopelagic fish in the world oceans is unknown. Biomass estimates published in the last 20 years range between 2 and 19.5 Gt. New acoustic estimates are over one order of magnitude above historic estimates based on net sampling^5,14–17^, challenging our understanding of gross ocean carbon production, major food chains and ecosystem carbon flow in these deep-water systems. Two main reasons leading to this uncertainty have been identified. First, mesopelagic fish species are difficult to fish due to high avoidance to experimental pelagic trawls^18,19^, potentially leading to an underestimation of their biomass. Second, the composition of the acoustic scatterers in the deep scattering layers may include other species than fish (e.g. siphonophores), potentially leading to an overestimation of the biomass of interest. To overcome this, a combination of trawl and multifrequency acoustic methodologies has been recommended for the estimation of mesopelagic fish abundance^20–22^.

The Bay of Biscay is in the southern region of the northeast Atlantic and shelters a large and diverse community of commercial species. To provide assessment and management advice on fish stocks, estimates of abundance are currently provided by expert groups^23^. However, to date there are no scientific surveys focused on the biomass estimation of mesopelagic species in the Bay of Biscay.

To convert acoustic data into biomass estimates, it is necessary to estimate the target strength (*TS*; dB re 1 m^2^), which is a measure of the amount of incident wave reflected by a single target^24^, and determine its relationship with fish length. When multifrequency acoustic data is available, it is often useful to measure the frequency-dependent difference in mean volume backscattering strength^25–29^ (ΔMVBS; dB re 1 m^−1^).

Global mesopelagic biomass estimations^5,14^ are reported from single frequency data (38 kHz), so no multifrequency analyses are available to date. However, using single frequency data can also be useful. For example, the use of low frequencies (i.e. 18 kHz) with long range and high signal to noise ratio^30^ allows collection of ecosystem information from the mesopelagic zone.

Few *TS* estimates have been reported for mesopelagic species using either *in situ*^31^ or *ex situ*^32^ measurements, modelling techniques^33^ or a combination of them^34,35^. For mesopelagic fish, resonance at frequencies ≤ 38 kHz is particularly pronounced^13,36^, however, resonance at frequencies up to 60 kHz have also been described for smaller swimbladder sizes of migrating^37^ and non-migrating fish^30^. In a study performed on pearlside in Masfjorden (Western Norway)^38^, frequencies around 60 kHz were essential to describe the size distribution of the pearlside population due to the small mean body size ranges observed (2.3–8 cm for mature individuals and < 2.9 cm for immature and male specimens). A recent study reported *TS* estimates at 18, 38, 120 and 200 kHz on this species in the Osterfjord in Norway^35^ and presented the first steps towards determining the *TS-*length relationship. Also, the main issues associated in determining *TS* values were identified there, offering the possibility to address them in subsequent studies such as this one.

Up to 95% of the backscatter of a swimbladder-bearing fish, is attributable to the swimbladder^39^ due to the density contrast between gas and water^40^. Changes in the volume or surface area of the swimbladder can influence the *TS* significantly^41,42^ and lead to considerable differences in abundance estimates. When studying *TS* versus length relationships of swimbladder-bearing fish, backscattering simulations derived from swimbladder morphology help to interpret and generalise the observed results. In the case of mesopelagic fish, these models are even more important due to the non-linearity between *TS* and length^43^. Pearlside is a physoclist species, that is, able to keep a constant volume of the swimbladder against pressure changes. However, its actual swimbladder volume compensation performance during the trawling process is not clear, hampering the interpretation of the observed swimbladder size at the surface. In fact, when modelling the swimbladder, there is lack of consensus in the literature on whether to consider pearlside as a physostome^13,15,35^ (the swimbladder volume obeys Boyle’s law) or as a physoclist^30,33,44,45^ species.

The main objective of this study is to provide estimates of *TS* to convert acoustic densities into biomass of pearlside. Acoustic-trawl data was used to determine the *TS* vs. length relationship estimates at 18, 38, 70, 120 and 200 kHz frequencies. Also, a comprehensive set of soft X ray images was used to determine the morphology of the swimbladder as well as its relationship with fish length. Theoretical *TS* values were simulated using an ellipsoidal approximation for the swimbladder. This work includes comparison of the model behaviour under different swimbladder tilt angle and contraction rate values, optimised by comparison with the experimental ones. According to the results, considerations about the physostome or physoclist behaviour of this species are provided as well as the most likely mean sizes of the swimbladder at the mean depth ranges of this study.

## Material and Methods

### Data collection

**Figure 1:**
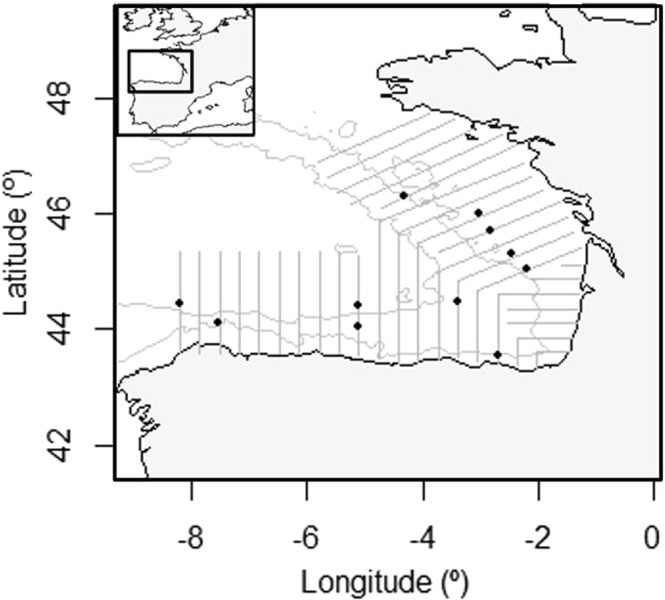
Area of data collection. Bay of Biscay map showing the transects (lines) and hauls (black dots) from all the years of study used for the target strength (*TS*) measurements.

Acoustic-trawl sampling was performed in September 2014, 2015, 2016 and 2017 in the Bay of Biscay during the acoustic-trawl survey JUVENA^46^ (Fig. 1). Additional sampling was done in year 2018 to collect biological pearlside samples for morphological measurements and to test the capture efficiency using different mesh size codends.

### Acoustic data

Acoustic records were collected onboard the research vessel Ramón Margalef (RM hereafter), using an EK60 scientific echosounder (Kongsberg Simrad AS, Kongsberg, Norway) with split-beam transducers of 18, 38, 70, 120 and 200 kHz, placed in a drop keel that reached a maximum depth of 6.75 m. All nominal beam widths were 7° except for the 18 kHz transducer, with a beam width of 11°. Pulse duration was 1024 µs with a ping rate of 0.7 s^−1^. The transducers were calibrated following standard procedures^47^ using a tungsten carbide sphere of 38.1 mm of diameter. The maximum sampling depth was 500 m.

### Biological data

Ground-truth trawl samplings were done with a Gloria HOD 352 pelagic trawl with 15 m of vertical opening, provided with a 10-mm mesh size (bar length) at the codend. Catches from these hauls were used to identify fish species and to determine their size distribution. Fishing trawls were performed between 15 and 300 m depth at a mean speed of 4 knots. Lengths were obtained from a random sample of each haul and measured to 0.5-cm standard length classes (*SL*; cm) onboard the research vessel.

**Figure 2:**
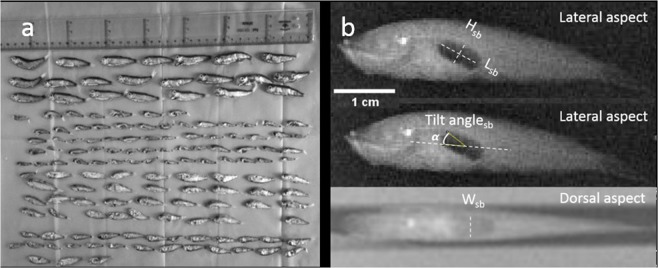
Biological samples. Spatial arrangement of fish samples for the X-ray session (**a**). Soft X-ray images of the lateral and dorsal aspects (**b**) of a specimen of *M. muelleri* (standard length, SL = 47 mm). A 1 cm scale bar was included.

A random subsample of 283 individuals was frozen in liquid nitrogen immediately after being captured and stored in individual plastic bags at −15 °C onboard the research vessel. Four months after being captured, frozen samples were carefully removed from the plastic bags and set in order by trawls. This was done in the laboratory in a temperature-controlled environment (0 °C) to minimise the damaging effect on the biological structures. The three cross-sectional dimensions of the swimbladder length (L_sb_; cm), height (H_sb_; cm) and width (W_sb_; cm) and the tilt angle (θ_sb_) were determined based on soft X-ray images (IntechForView CR system) of the lateral and dorsal aspects of the fish (Fig. 2). Only the specimens with undamaged swimbladders were considered for the measurements (i.e. there were some cases in which these were absent or disfigured and bubbles of air were visible elsewhere from the swimbladder). The dimensions of the detector plate were 430 mm × 350 mm with a pixel size of 86 µm. Samples were located at a distance of 1 m from the source and exposed to 40 kV per 1.6 mA/sec.

### Catchability of small length classes

To test whether the mesh size codend was able to efficiently capture the whole length distribution of the pearlside population, we used two different codends on the same model of pelagic trawl. One codend had the 10 mm minimum mesh used for the samples involved in the *TS* analyses. The other codend had a gradual mesh size, ranging from 8 to 2 mm, specially designed to target micronekton species. In total there were 21 positive hauls of pearlside for the experiment; from these, 13 were done with the small mesh and 9 with the large one. The experimental procedure consisted of measuring the length of 100 individuals from each trawl to compare the length distributions obtained with both gears using statistical analysis of variance (ANOVA).

### Ethics statement

All samples were collected under permission of the corresponding local authorities: *Ministère des affaires étrangéres* (France), *Vice. de Agricultura, Pesca y Politicas Alimentarias* (Basque Country) and by the Spanish Government “Administración del Estado, Ministerio de Agricultura, Alimentación y Medio Ambiente, Secretaría General de Pesca”. All methods and research conducted in this study were carried out under the guidelines provided by article 5 of the European Convention for the protection of vertebrate animals used for experimental and other scientific purposes (Cons 123 (2006) 3)^48^ in accordance with AZTI’s policies.

### Data analysis

#### Single frequency analyses: spatial analysis and biomass estimation

The acoustic backscattering at 38 kHz collected during the transects was echointegrated annually by 0.1 nmi (elementary distance sampling unit or EDSU) per ~50 m bins, to a maximum depth of 500 m. This part of the survey strategy^46^ consisted in providing spatial distribution and biomass annual estimates of several species at a single frequency (38 kHz). Acoustic energy was first cleaned from unwanted signals and then echointegrated using a threshold of −60 dB. The software used for this purpose was Movies + (developed by Ifremer, France). The nautical area scattering coefficient (*s*_*A*_; m^2^ nmi^−2^) was allocated by species and size according to the hauls and the echogram typology. It was then used to obtain the mixed species echointegrator conversion factor^24^. The *s*_*A*_ allocated to pearlside was used to produce spatial distribution maps and vertical profiles, as well as to examine the effects of daily vertical migration (DVM). Finally, the abundance in numbers was obtained after dividing *s*_*A*_ by the mean backscattering coefficient of pearlside and multiplying by the mean weight and EDSU to obtain the annual biomass in the studied area.

#### Multifrequency analyses

**Figure 3:**
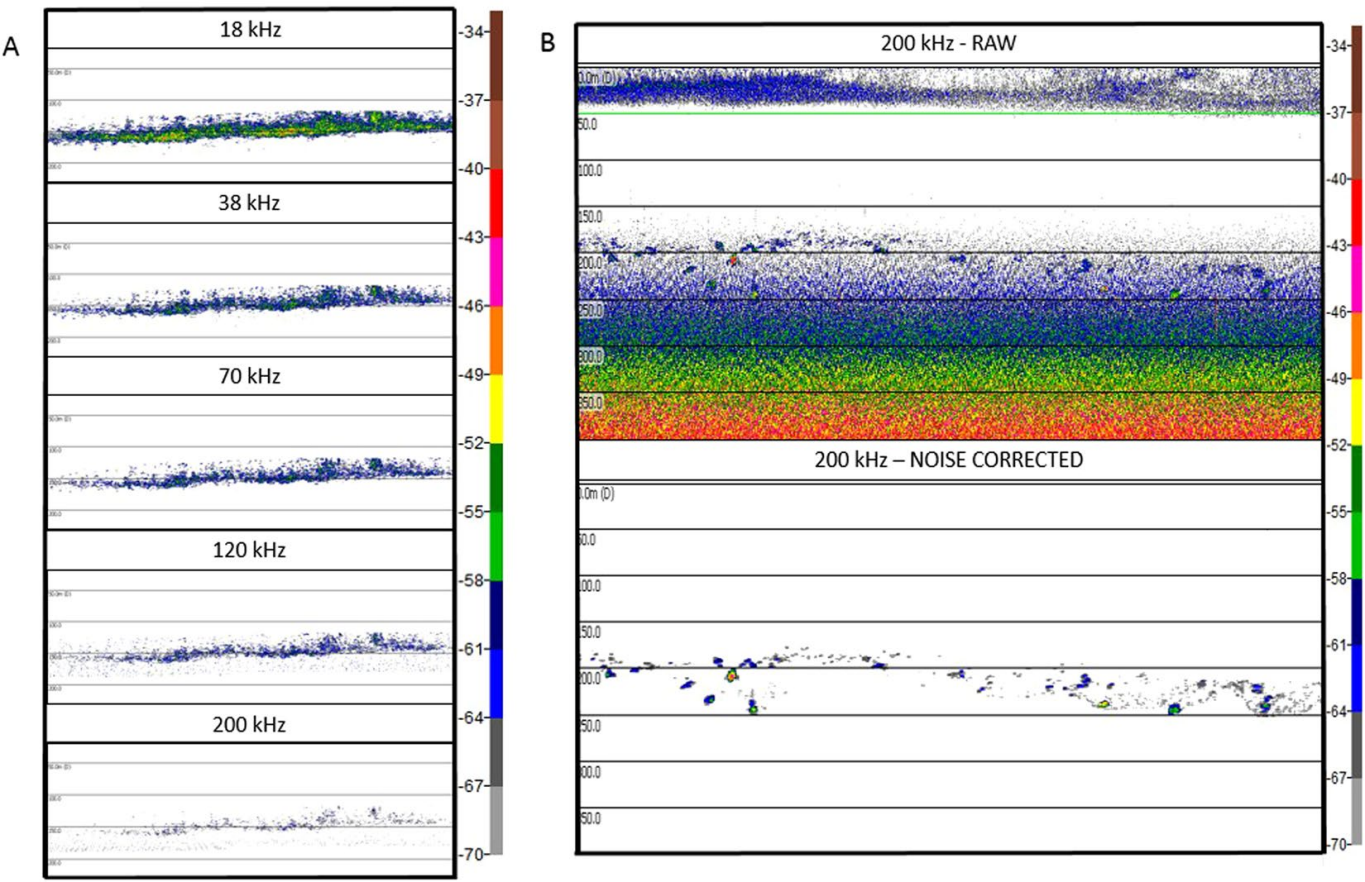
Example echograms. Echograms showing the typical pearlside multifrequency scattering layer (**A**) and the background noise correction applied to the 200 kHz frequency, at depths greater than 100 m (**B**).

Multifrequency analysis was done on acoustic data collected from hauls with more than 90% of the catch being pearlside (Fig. 3A) using Echoview software^49^. The deepest trawl was performed at a mean depth of 163 m (Table 1). Due to the range limitation of the high frequencies, background noise that registered below 100 m at 200 kHz was removed (Fig. 3B) following the techniques described by De Robertis and Higginbottom^50^ (cells of 20 pings by 5 samples, smoothed via 5 × 5 convolution into the background noise removal operator, with maximum noise of −125 dB and minimum signal-to-noise ratio (SNR) = 1.

**Table 1:** Details of the pelagic trawls used for the mean volume backscattering strength (MVBS; dB re 1 m^−1^) differences and target strength (TS; dB re 1 m^2^) analyses.

Trawl	Date	Time	Lat	Lon	Depth	Catch	Catch%	Length
		GMT	°	°	m	g	% (g)	cm (±sd)
9010	08/09/2014	22:13	44.06	−5.10	70	2713	91	3.0 ± 0.2
9002	31/08/2015	9:05	44.46	−8.20	75	848	100	4.3 ± 0.6
9006	05/09/2016	12:31	44.05	−5.10	153	950	100	2.6 ± 0.3
9012	08/09/2016	12:10	43.54	−2.70	163	13.3	97	4.9 ± 0.9
9020	22/09/2016	12:21	45.70	−2.82	106	1300	100	4.0 ± 0.5
9006	06/09/2017	19:43	44.10	−7.52	120	1500	36^*^	2.9 ± 0.5
9012	16/09/2017	19:00	44.48	−3.38	51	92000	37^*^	2.7 ± 0.4
9013	17/09/2017	13:45	45.05	−2.20	120	5850	100	4.7 ± 0.8
9015	18/09/2017	16:00	45.21	−2.45	114	145127	97	3.6 ± 0.6
9017	20/09/2017	9:50	45.94	−3.03	105	92000	100	3.8 ± 0.5
9031	27/09/2017	18:03	46.30	−4.31	55	13.5	97	4.1 ± 0.4

Time: (UTC + 2), start times of echogram section, Depth: mean depth of the echointegrated section, Catch (estimated catch of species), Catch%: percentage of the catch being pearlside and Length: mean length (±standard deviation) of the specimens in the catch. *Night hauls with mixed composition of krill and pearlside. The echogram sections used for the analyses corresponded to daytime (2 hours earlier than the trawls approx.), when the stratification of these species was evident and a layer of pure pearlside could be used for processing.

Frequency dependent dB difference: Echointegrations were done using Echoview^49^ over cells of 50 m vertical x 0.1 nautical mile with a −70 dB minimum threshold; subsequent analyses were performed in R software^51^. ΔMVBS between frequencies is often used to discriminate between scattering groups^25–29^. In this study, ΔMVBS was calculated for all frequencies using 38 kHz as the reference frequency. All averaging was performed in the linear domain and converted back to the logarithmic scale.

*In situ* TS analysis: *In situ TS* values were derived from echosounder data using the Echoview single target detection algorithm for split beam echosounders^52^. A −70 dB minimum threshold was applied with a pulse determination level of 6 dB. The minimum and maximum normalised pulse lengths were 0.7 and 1.5, respectively, the maximum beam compensation applied was 6 dB and the maximum standard deviation of minor and major axis angles was 0.6 degrees.

To reduce the bias caused by a poor signal to noise ratio towards the edge of the acoustic beam, the density of target detections was examined within each degree ring of the beam. A 3° cut-off angle filter was used to reject target detections that were in lower densities (corresponding to −3 dB off axis)^35,53^.

A high-density filtering method^54^ was applied to reduce the multiple target bias^55^. This was necessary since small pelagic fish occur in high packing densities that are likely to prevent the successful detection of single target echoes^56^. Target strength measures were rejected when the number of fish per acoustic reverberation volume (N_v_), calculated following the procedure described by Ona and Barange^55^, surpassed an empirically-determined density threshold. This was located at the inflection point of the number of targets per sample volume (T_v_) on the N_v_, where the target density is such that multiple target echoes are likely to be produced^54^. The effect of the horizontal measurement scale on the threshold value was examined by filtering the *TS* detections by the different thresholds calculated at intervals of 100, 50, 25, 10 and 5 pings (1 ping ≈ 1.8 m) per 5-m depth cells.

After being filtered for the SNR and multiple targets, the *TS* dataset was used to estimate *b*_20_ from Eq. (1) at five frequencies (18, 38, 70, 120 and 200 kHz) by the least-squares fitting procedure described in MacLennan and Menz^57^. The filtered *TS* dataset was fit to a normal distribution derived from the fish size histogram of the catches (modelled *TS* distribution assuming 20 log *SL*) to evaluate the mean, standard deviation (*SD*) and *b*_20_ of the best fit, given by the coefficient of determination (*R*^2^).

1 $$TS=20\,log(SL)+{b}_{20}\cdot $$

#### Acoustic scattering models

Since gas-filled swimbladders reflect 90% or more of the backscattered energy^39^, only these were considered for modelling the backscattering strength. The effects of depth and size on the swimbladder target strength were analysed using a scattering model that applied an ellipsoidal approximation for the swimbladder^58–62^.

The semi-major (*a* = L_sb_/2) and semi-minor axes in the lateral (*b* = H_sb_/2) and dorsal (*c* = W_sb_/2) aspects were used to calculate the equivalent sphere radius *a*_*esr*_^63^:

2 $${a}_{{\rm{esr}}}={(abc)}^{1/3}\cdot $$

All the equations used in this study, as well as the environmental and material properties, were adopted from Andreeva^58^ and Love^60^ (see Supplementary Table 1). The sequence in which the different equations of the model were used followed the same structure as in Scoulding *et al*.^35^.

Although pearlside is a physoclist species^64^, different assumptions related to the depth dependence of swimbladder volume were compared. (1) Swimbladder dimensions were independent of depth due to volume compensation associated with physoclist species^30,33,36,44^; thus, we assume no effect of Boyle’s law. (2) A pressure-induced volume reduction of the swimbladder was considered according to Boyle’s law^13,15,35^ by which the dimensions at the fishing depth were expected to be smaller than those observed at the surface. In this case, the following model was used:

3 $${\sigma }_{z}={\sigma }_{0}{(1+\frac{z}{10})}^{{\rm{\alpha }}},$$

where *σ*_*z*_ is the backscattering cross-section at depth *z*, *σ*_0_ at the surface and 𝛼 is the estimated contraction rate parameter (−0.67 for a free ellipsoid)^65^. (3) This assumption accounted for the mechanical stress of the fish derived from the trawling process, where 𝛼 was treated as a floating parameter of values ranging from 0 to −0.67. Values for mean ($\bar{\theta }$) and standard deviation (***σ***_θ_) of tilt angle were obtained from the X-ray images. (4) The whole space of combined parameters was explored, using γ, $\bar{\theta }$ and ***σ***_θ_ as floating parameters. Except for the third variant of the model, in which the tilt angle parameters were determined from the RX images, the other three assumptions explored normal distributions with mean values ranging from 0–70° and standard deviations of 0–30°.

The Akaike information criteria (AIC) was used to select the best variant of the model because it takes into account the goodness of fit of the model and penalises the use of optimised parameters over the use of parameters with fixed values.

4 $$AIC=nlog(S{S}_{res})+2(p+1)-nlog(n)$$

where *n* is the number of observations and *p* is the number of floating parameters used. The optimal model was then used to interpret the actual swimbladder behaviour of pearlside.

## Results

### Biological sampling

**Figure 4:**
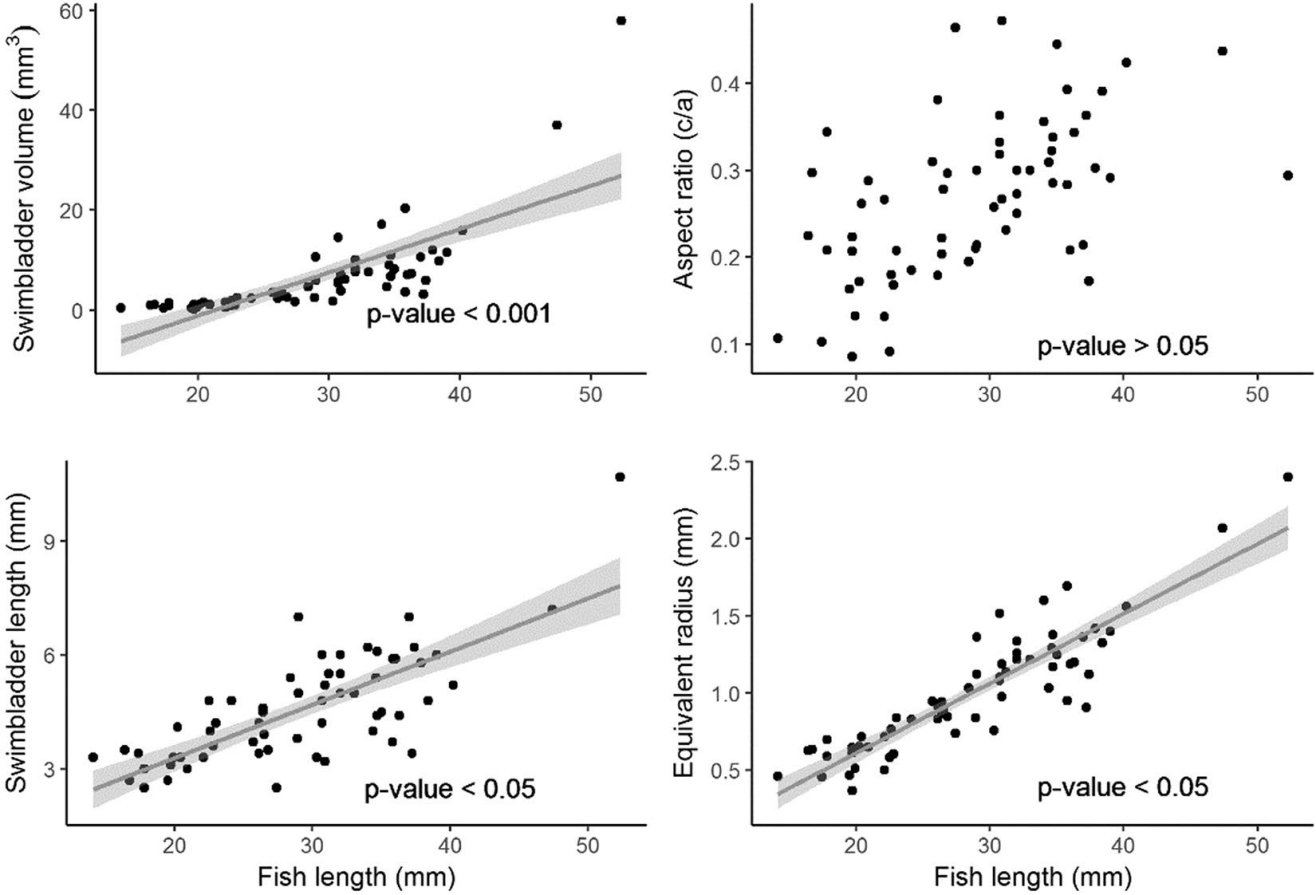
Swimbladder morphological measurements. Relationship between standard length (mm) and the swimbladder volume, aspect ratio (Ɛ = c/a), swimbladder length and equivalent radius of the 63 specimens with gas-filled swimbladders. The shadowed area represents the 95% confidence intervals.

A total of 11 trawls where *M. muelleri* was the dominant species were used for the analyses (Table 1). Krill *Meganyctiphanes norvegica* contributed on average 3.6% of the total numbers, while squid (*Loligo vulgaris*), salps (*Salpasalpa*) and jellyfish (*Rhopilema spp*.) contributed to the catches to a lesser extent. Standard length distributions of pearlside ranged from 2.6 ± 0.3 cm to 4.9 ± 0.9 cm. A total of 63 individuals with apparently undamaged swimbladders were finally used for the morphological measurements. The pearlside swimbladder appeared as a regular-shaped single-chamber ellipsoid with a long (*a* = L_sb_/2) and short (*b* = H_sb_/2) lateral semi-axis and a short dorsal semi-axis (*c* = W_sb_/2) and an average tilt angle of 24° ± 7° (Fig. 2, Table 2). Results indicate that for an increase in fish length, there is an increase in swimbladder volume (r^2^ = 0.6, p < 0.001), length (r^2^ = 0.07, p < 0.05) and equivalent radius (r^2^ = 0.6, p < 0.05) (Fig. 4). As for the aspect ratio, data suggest a positive correlation with fish length, although this was not significant (r^2^ = 0.02, p > 0.05).

**Table 2:** Results of the morphological measurements of the swimbladder (n = 63).

	Symbol	Units	Range	Mean ± SD
Standard body length	L _f_	cm	1.41–5.23	2.87 ± 0.78
Length	L_sb_	cm	0.25–0.98	0.45 ± 0.14
Height	H_sb_	cm	0.07–0.35	0.20 ± 0.07
Width	W_sb_	cm	0.03–0.31	0.12 ± 0.06
Dorsal area	A_sb.D_	cm^2^	0.006–0.17	0.05 ± 0.03
Lateral area	A_sb.L_	cm^2^	0.01–0.18	0.06 ± 0.04
Long lateral semi-axis	a	cm	0.13–0.49	0.22 ± 0.07
Short lateral semi-axis	b	cm	0.03–0.16	0.08 ± 0.02
Short dorsal semi-axis	c	cm	0.01–0.15	0.06 ± 0.03
Swimbladder ratio	a/L_f_	—	0.05–0.12	0.08 ± 0.02
Aspect ratio	Ɛ	—	0.09–0.47	0.26 ± 0.09
Tilt angle	θ	°	11.4–43.6	24 ± 7

#### Capture efficiency vs mesh size experiment

The mean ± standard deviation body length of the fish captured with the 8 to 2 mm mesh was 3.3 cm ± 0.8 cm whereas for the 10 mm mesh it was 2.7 cm ± 0.7 cm, with the minimum sizes caught being ~1.5 cm in both cases (N = 1201). To further assess this, in one particular site, we were able to repeat two trawls consecutively, targeting the same aggregation using both mess sizes. In this case, the mean sizes were 3.5 cm ± 0.4 cm and 3.6 cm ± 0.4 cm for the 8–2 mm and 10 mm mesh sizes, respectively, and the statistical tests provided non-significant differences between means (p > 0.05). According to this result both trawls seem equally able to perform sampling of small sizes in the range of this study and hence the biological sampling for the *TS* analysis was considered to be unbiased and representative of the true pearlside size distribution.

### Spatial distribution patterns of Mueller’s pearlside in the Bay of Biscay

**Figure 5:**
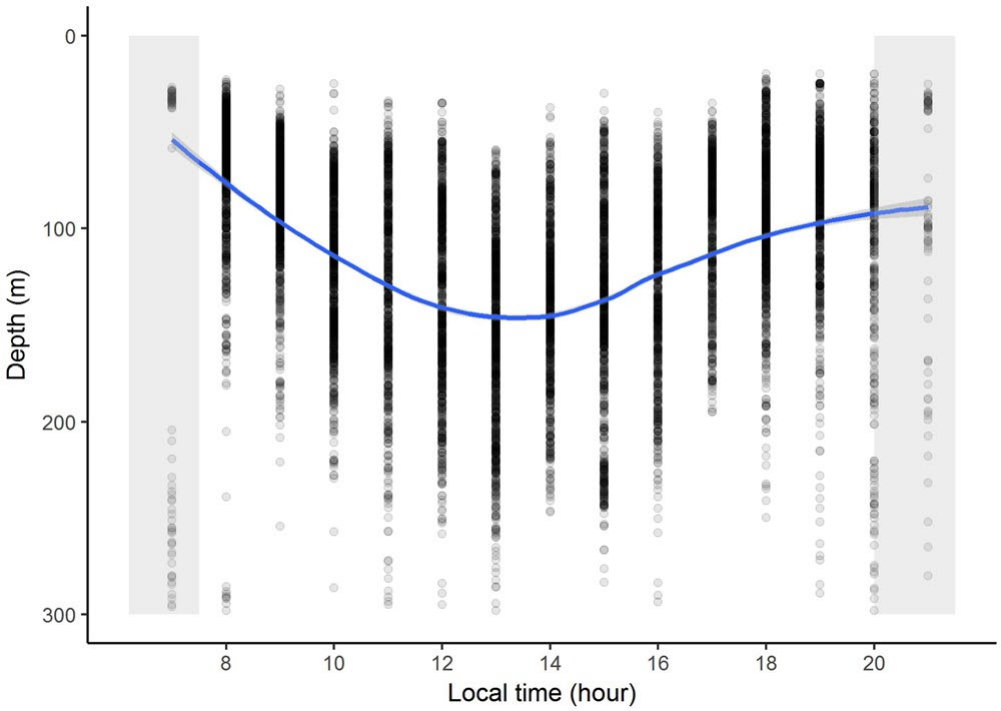
Vertical migration. Diurnal vertical migration patterns of *M. muelleri* with mean depth (m) plotted against local time of day in hours. The density of points is proportional to the nautical area scattering coefficient (*s*_*A*_; m^2^ nmi^−2^). Loess smoother represented as solid line.

Mueller’s pearlside was predominantly found off the shelf or at the outer part of the continental shelf, although it reached the 100 m isobath on the French shelf. Its vertical distribution during daytime ranged from 50 m down to the maximum depth sampled in this study (500 m). The location of the acoustic detections of pearlside in the water column varied with time being on average about 50 m shallower during nighttime (Fig. 5).

### Frequency dependent dB difference

**Figure 6:**
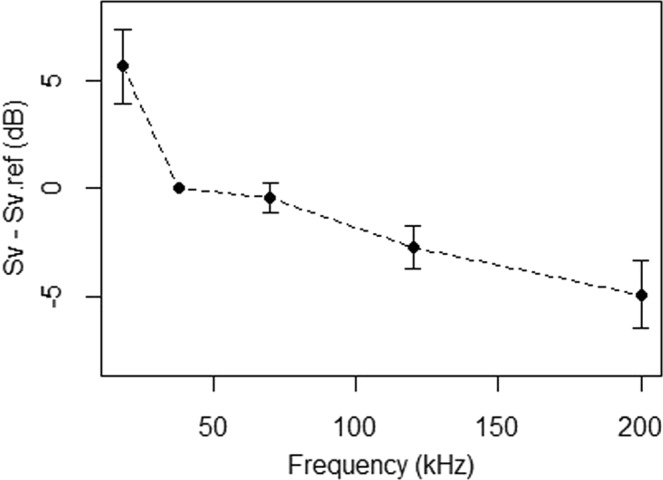
Averaged *in situ* dB difference ΔMVBS_38_ of pearlside. A general decreasing trend was observed with increasing frequency. Error bars indicate 95% confidence interval.

Pearlside ΔMVBS_38_ showed a general decreasing trend towards high frequencies. The observed pattern described the highest difference at 18 kHz with a sharp decline towards 38 kHz, consistent with the presence of a resonance peak at frequencies below 38 kHz. There was an approximately similar response at 38 and 70 kHz and a final decay for the 120 and 200 kHz frequencies (Fig. 6).

### *In situ* TS

**Figure 7:**
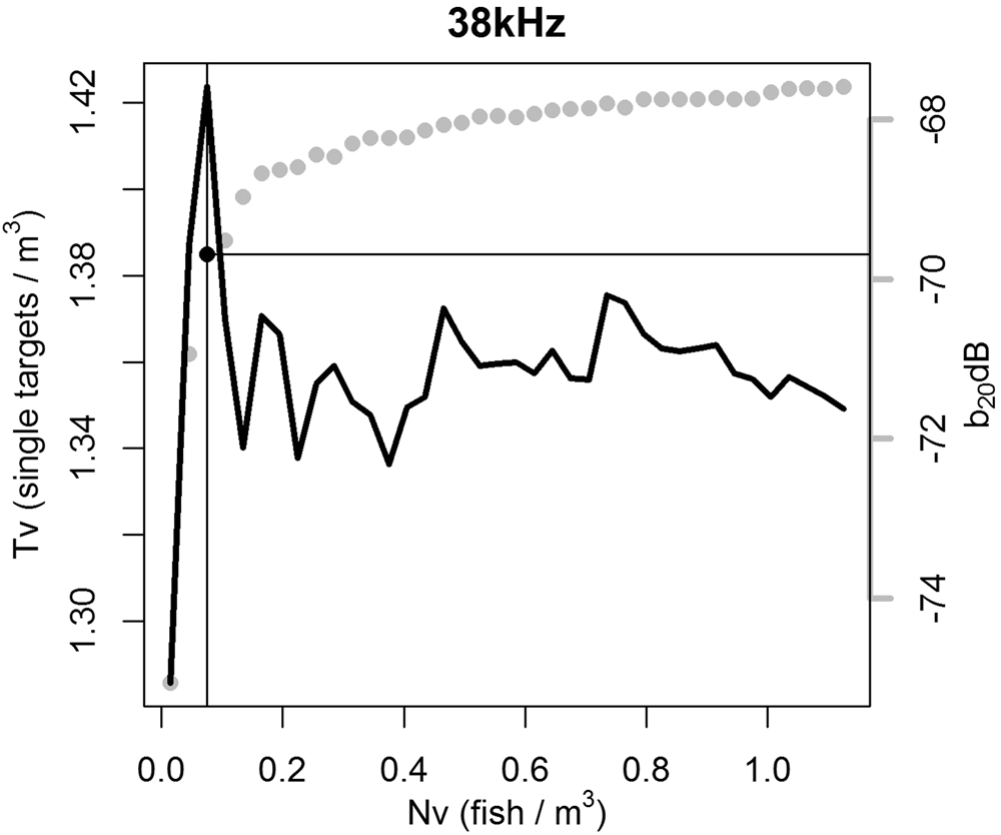
Example of the number of targets per sample volume (T_v_) against number of fish per acoustic reverberation volume (N_v_) at 38 kHz. Grey points are the b_20_ values averaged for every N_v_ threshold value. Black point indicates filtered b_20_ value at *T*_*v*_*/N*_*v*_ inflexion point, that corresponds to a 0.075 N_v_ threshold.

Even if the N_v_ values varied within the scale of measurement, the averaged *TS* values were constant regardless of the grid size, showing differences of less than 0.2 dB within scales. The smallest scale size (5 pings × 5 meters) was chosen for the N_v_ threshold determination. The point of inflection of the number of T_v_ on the fish number N_v_ (Fig. 7) was observed at threshold values of 0.12, 0.07, 0.16, 0.06 and 0.04 fish per m^3^ at 18, 38, 70, 120 and 200 kHz frequencies, respectively, meaning that only cells that passed those thresholds were retained for subsequent analysis. The filtered *TS* datasets consisted of 109, 154, 578, 255 and 158 targets at each respective frequency, on which the *b*_20_ fitting procedure was based.

**Figure 8:**
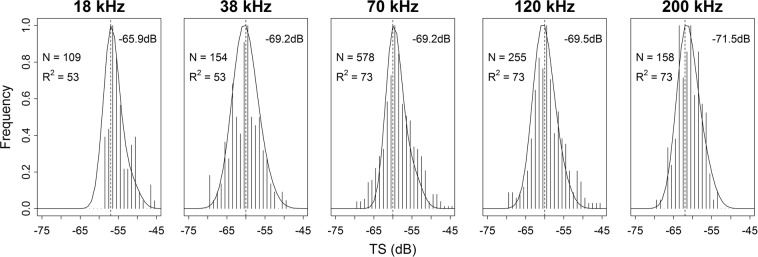
Predicted and observed *TS* fitting procedure. The filtered *TS* dataset (black vertical solid lines) was fit with a normalized length distribution (solid curve) to evaluate the mean (dashed vertical line), standard deviation (2, 3, 2, 2.5 and 2.5 for 18, 38, 70, 120 and 200 kHz, respectively) and *b*_20_ (topright corner of each panel) of the best fit, given by coefficient of determination (*R*^*2*^) of observed versus modelled *TS* distributions. N stands for the number of targets that passed the filtering process and were used in the optimization.

The best-fit *b*_20_ values derived from the N_v_-filtered *TS* and SL distributions were −65.9, −69.2, −69.2, −69.5 and −71.5 dB for the 18, 38, 70, 120 and 200 kHz, respectively, with coefficients of determination (R^2^) ranging from 53 to 73% (Fig. 8). These *TS*-length relationships correspond to the depth range of the filtered dataset (17–143 m) and standard fish length ranging from 2.7 to 4.3 cm.

### Biomass estimation

The biomass of pearlside in the Bay of Biscay was calculated with single frequency data registered at 38 kHz over the four years analysed in this study. It followed a decreasing trend from 2014 to 2016, but reached maximum numbers in 2017. The minimum and maximum estimates were 70.7 and 161.7 thousand tons in years 2016 and 2017, respectively (Table 3).

**Table 3:** Time series of biomass estimation of pearlside in the Bay of Biscay.

Year	<s_A_>	Area	Mean weight	Mean length	Biomass @ 38 kHz	CV
	(m^2^nm^−2^)	(nm^2^)	(gr)	(cm)	(Tn)	(%)
2014	309.3	21,073	0.51	3.42	142,242	30
2015	630.79	8,663	0.58	3.96	127,447	35.3
2016	348.96	7,189	0.36	3.44	70,784	68.2
2017	511.30	13,313	0.53	3.68	161,713	35.7

### Acoustic scattering model

**Figure 9:**
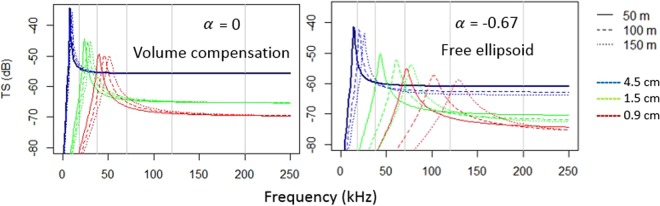
Scattering model simulations. Resonance scattering model behavior for simulations of different sizes and depths, considering swimbladder contraction rates 𝛼 = 0 (left) and 𝛼 = −0.67 (right). In these theoretical simulations, broadside incidence (θ = 0°) was assumed.

The general behaviour of the backscattering model used was illustrated by simulating the *TS-*length and *TS-*depth relationships for swimbladder contraction rates 𝛼 = 0 and 𝛼 = −0.67 (Fig. 9). Regarding the size effect, modelled *TS* values decreased with decreasing swimbladder size, but the resonance frequency increased. The effect of size on the resonance frequency was clearly seen when 𝛼 = −0.67, but smaller when 𝛼 = 0 (estimated to be below 50 kHz for all the examined sizes). The effect of depth on the resonance frequency was minimal when 𝛼 = 0, but clearly observed when 𝛼 = −0.67. Maximum *TS* values at resonance decreased with depth, having a major effect when 𝛼 = −0.67. Depth variations produced major changes on smaller swimbladder sizes.

When 𝛼 was set to 0 (no pressure effect) and the tilt angle was used as a floating parameter, the optimised tilt angle was 70° ± 5 (Table 4a). The lowest AIC value was achieved when using a fixed 𝛼 = −0.67 (Boyle’s law effect), and the mean tilt angle (*θ*) that minimised the distance between the modelled and experimental *TS* values followed a normal distribution with a mean of 10° ± 5 (Table 4b). The model simulation that assumed the measured *θ* ± ***σ***_*θ*_ from the RX images (24° ± 7), produced an optimised contraction rate of −0.66 (Table 4c). The highest AIC value was obtained when the three variables were treated as floating parameters, and the whole space of combinations among parameters was evaluated with the ranges defined above (Table 4d).

**Table 4:** Performance comparison (AIC, Akaike Information Criteria) of the different backscattering model variants tested. Mean depth and fish length averaged from filtered dataset:

Swimbladder contraction rate (γ)		Mean tilt angle (θ)	SD tilt angle (*σ*_θ_)	Number of optimized parameters (*)	AIC
(a)	−0.66*	24**	7**	1	16
(b)	0	70*	5*	2	15
(c)	−0.67	10*	5*	2	8
(d)	−0.62*	65*	10*	3	19

*optimized parameters in each model variant.

**values measured from the RX images.

**Figure 10:**
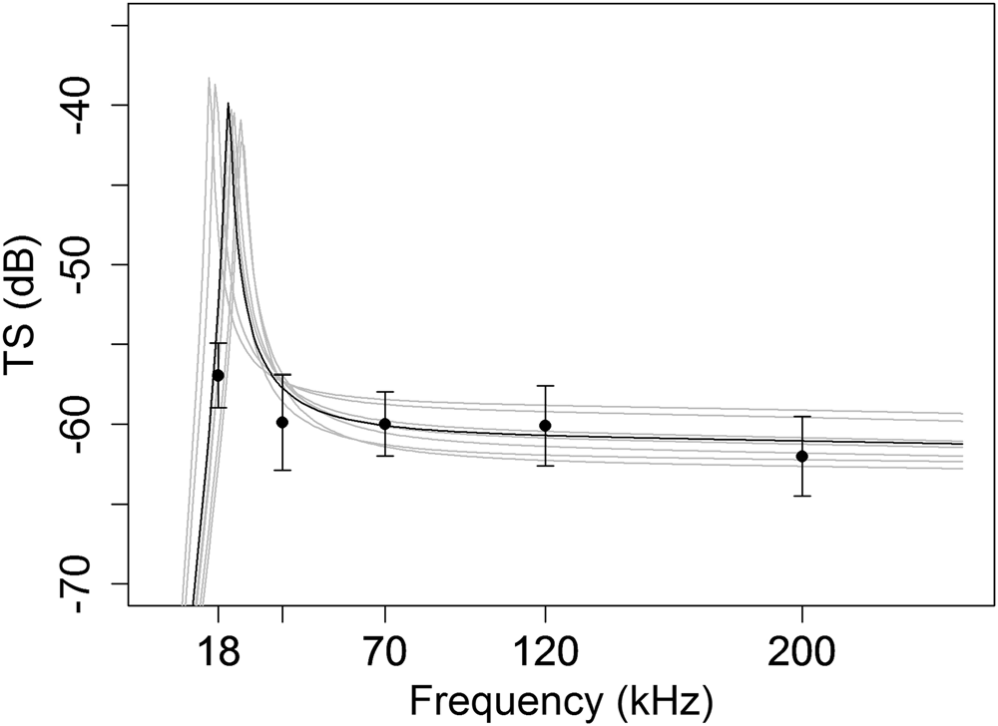
Model vs filtered *in situ* TS data. Optimal model (𝛼 = −0.67 and *θ = *10° ± 5) plotted for frequencies from 0 to 250 kHz using mean depth 84.5 m and mean length 3.68 cm (black line). Additional curves show the model behavior using depths and standard lengths associated to the trawls used in the study (grey lines). Black dots are the *in situ* filtered *TS* values with error bars showing the standard deviation from the mean values.

The optimal model (𝛼 = −0.67 and *θ* = 10° ± 5) was plotted for a range of frequencies from 0 to 250 kHz, for a mean depth of 84.5 m and mean *SL* of 3.68 cm (Fig. 10). Additional curves were included using the mean depth and length from all the trawls used in this study (in grey). The *in situ* filtered *TS* data at the five frequencies of study (black points) fit the model curve closely (Fig. 10).

## Discussion

This study provides the basic elements necessary to estimate the biomass of an important mesopelagic species, *Maurolicus muelleri*, as well as an estimation of its biomass during the years of study. Although pearlside has its mean vertical distribution within the epipelagic zone, it can reach more than 400 m depth during the day. This variation in vertical distribution seems to be dependent on water temperature and oxygen availability^66^. There were two sources of information used for these analyses: multifrequency acoustic data associated with pure or almost pure monospecific catches of pearlside, and single frequency data allocated to layers homogeneous in species and size composition. Acoustic data was subjected to a thorough cleaning and filtering process by removing the two main sources of bias that could affect the mean *in situ TS* values^55^: a low SNR ratio and the acceptance of multiple targets as single target detections. The latter is derived from the suboptimal performance of the single target detection algorithm that accepts overlapping echoes, affecting the *TS* distributions and, therefore, biasing the mean *TS*.

Following a similar procedure as in Boyra *et al*.^67^, the performance of several methods was tested as part of a preliminary analysis of data: variation of the maximum standard phase deviation^52^, a multiple-frequency method to retain only targets detected by more than one frequency^68,69^ and a high-density filtering method using an empirically-determined density threshold^54,70^.

These methods were evaluated according to their filtering potential and the number of targets that were available for the subsequent *b*_20_ estimation. From the applied methods, the standard phase deviation did not affect mean *TS* values (varied less than 0.5 dB) and was thus discarded. The multiple frequency and the high-density filtering methods provided rather similar results (differing in 2, 0.8, ~3, ~3 and 2.5 dB at 18, 38, 70, 120 and 200 kHz frequencies, respectively). However, since the maximum distance between the spatial coordinates of the detections at different frequencies was larger than the typical size of the target species, the multiple frequency method was not considered reliable. The authors therefore focussed on the high-density filtering method. Using this method, the mean *TS* value was independent of the horizontal scale used, hence proving robust results. Also, based on the methodology applied, the mean *TS* value obtained was independent from the initial *TS* value used for the N_v_ determination as this did not affect the location of the inflection point relative to the “x” axis of the plot (Fig. 7).

After the filtering process, there were sufficient target detections to compare the observed *TS* histogram with the size distribution of the insonified fish^57^ (Fig. 8). Further filtering was not considered necessary since it did not vary the mean *TS* estimate and the remaining targets were insufficient for a subsequent *b*_20_ optimisation.

The ΔMVBS_38_ of pearlside showed a general decreasing trend (Fig. 6), which is in agreement with the presence of a resonance frequency below 38 kHz. This fits with the expected response of the observed fish sizes (>2.5 cm, Fig. 9). The largest variability in the ΔMVBS_38_ was observed at 18 kHz, probably due to the higher slope caused by the proximity to the resonance frequency. This decreasing trend agreed with previous works on this^35^ and other bladdered species^71^. The most used frequencies in multifrequency studies are 18, 38, 120 and 200 kHz. However, in this study, the 70 kHz was also included.

The present results agree with *TS* estimates of pearlside from previous studies (Table 5): values ranging from −60.4 to −52.5 dB at 38 kHz were reported for a total length of 4.5–5.7 cm at 10–50 m depth^72^, −70 to −50 dB was estimated for 2–4 cm specimens between 10–60 m depth^31^. Target strength estimates for 2.3 and 3.5 cm specimens at 20–64 m depth varied from −60.3 to −60.8 dB at 38 kHz according to Scoulding *et al*.^35^. This is 0.5–1 dB higher than our results. In comparison with the reported multifrequency *TS* estimates of that study, our results were inside their range at 18 kHz, but 2–4.5 dB higher at high frequencies. One possible explanation for this discrepancy could be due to the effect of tilt angle on high frequencies^33,35^. Variable fish behaviours during the *TS* measurements could result in a high variability of tilt angles. However, our results imply a smaller difference than studies analysing other similar species^32^.

**Table 5:** Summary table with relevant *TS* estimates published in the last 20 years.

Reference	Specie	Depth (m)	Length (cm)	TS (dB)				
				18 kHz	38 kHz	70 kHz	120 kHz	200 kHz
This study	*M. muelleri*	17–137	2.7–4.3	−56.9	−59.8	−59.9	−60	−62
Scoulding *et al*. (2015)	*M. muelleri*	20–64	3.5	−53.6	−60.8	—	−62.9	−66.4
Scoulding *et al*. (2015)	*M. muelleri*	20–64	2.3	−57.1	−60.3	—	−62	−65
Benoit-Bird and Au (2001)	*Myctophids*	0–200	3.7–6.1	—	—	—	—	−58.8
Sawada *et al*. (2011)	*D. Theta*	150	5.4–5.5	—	—	−55.8	—	—
Torgersen and Kaartvedt (2001)	*M. muelleri*	10–60	2–4	—	−70 to −50	—	—	—
Yoon *et al*. (1999)	*M. Muelleri*	10–30	4.5–5.7	—	−60.4 to −52.7	—	—	—
Yoon *et al*. (1999)	*M. muelleri*	30–50	4.5–5.7	—	−59.2 to −52.5	—	—	—

The derived *TS* versus length relationships in this study show consistency with the positive and significant correlation found between standard fish length and volume of swimbladder (Fig. 8). This represents a step forward compared to a recent study of the same species^35^ where a consistent *TS*-length relationship was not achieved because no clear relationships were found between standard length and swimbladder volume.

One of the remaining uncertainties about pearlside is the major effect that changes in depth associated with capture may have on swimbladder size. It is commonly assumed that pearlside, being a physoclist species, can absorb and secrete gas from the swimbladder to maintain a constant buoyancy while moving through the water column^24^. However, it remains unclear whether pearlside can compensate the swimbladder volume during the trawling process. The swimbladder can be overexpanded and even damaged due to decompression^73^ or mechanical stress. When modelling swimbladder backscattering, some studies used smaller sizes than those measured at the surface, compressed according to Boyle’s law^13,15,35^. However, other studies used swimbladder dimensions measured at the surface and therefore considered pearlside as strict physoclists^30,33,36,44^. Furthermore, it remains unknown if pearlsides allow swimbladder gas to expand and compress with changes in depth in undisturbed conditions^74^. Additionally, gas volume measurements at the surface are problematic due to the differences in pressure and temperature conditions between the surface and the depth of capture^43^. To address this issue, we simulated swimbladder backscattering response under different ranges of fish length, depth, tilt angle and swimbladder contraction rates. We then compared the simulated *TS* values to the experimental ones. The best model fit was achieved when a free ellipsoid was simulated (i.e. no volume compensation) with an incidence angle of 10° ± 5 (Table 4b). These results support the hypothesis that fish in the process of dying cannot compensate for the rapid pressure changes derived from capture. Therefore, the swimbladder volume seems to obey Boyle’s law^13,15,35^. Even if the physiological mechanisms lying behind these results may be more complex, it can now be assumed that the acoustic backscatter of captured pearlside must be modelled under a constant-mass assumption. Therefore, our modelling results support the hypothesis that the equivalent radius of the swimbladder at the mean depth of the trawls (84.5 m) would be 47% smaller than that measured at the surface (Table 2), which implies a 90% reduction of swimbladder volume.

This study shows significant positive correlations between the length of pearlside and three of the studied morphological parameters (swimbladder length, volume and equivalent sphere radius) (Fig. 4). The swimbladder volume relationship with fish length was already assessed in a previous study^35^, although no clear relationship was reported. The positive correlation between aspect ratio and fish length suggested that the swimbladder tends to be more elongated for smaller individuals. On a study focussed on similar species (*M. japonicus)*^33^, positive correlations were described for swimbladder length and equivalent radius with fish length. However, no correlation between the aspect ratio and fish length was reported. The swimbladder mean tilt angle measured from the X-ray images (24° ± 7) fit within the range of values published for similar^33^ and same species^35^, being 0–24.8° and 0–55°, respectively.

The optimisation of the model parameters produced a mean tilt angle of 10° ± 5. Therefore, one might conclude that the mean orientation of fish that best explains our data is −14° (±9°) (obtained from subtracting the tilt angle of the swimbladder from the modelled optimal tilt angle). This would suggest that fish from the hauls used in this study were predominantly exhibiting a downwards swimming behaviour. However, mesopelagic species and in particular pearlside, can adopt a wide range of orientation angles along the diel cycle performing DVM^13,75,76^. This behaviour has been described as response to diverse hypothesised adaptive values^76^ including predator avoidance^77,78^, optimal temperatures^79^ and improving feeding conditions^80^. Other factors that may induce variations of the tilt angle are time of day and time of year of data collection^70^, swimming behaviour^41^, schooling density^39,81^ and dispersion or position^65^ in the water column. This suggests that the variability of data belonging to different trawls, as done in this study, might be greater than reflected here. However, even if the effect that this variability has on the modelled *TS* increases with frequency and size, it is minimal at lower frequencies^33,35^. This implies a minimal effect on the frequency typically used for biomass estimation (38 kHz).

Mesopelagic fish are known to avoid or escape from fishing trawls^18,19,22,82^ which might bias the length distribution of the population^83–85^. In this study, the result of the mesh size experiment proved that the fishing gear used was able to sample size ranges found in our area of study (1.5–6.5 cm). However, it is recommended to test the capture efficiency of each sampling area before performing studies on mesopelagic fish and, if significant differences are found, minimise the associated bias by applying a capture efficiency correction factor^85^.

Absolute abundance estimates are very sensitive to the *TS* value and are therefore a major source of uncertainty for such estimations^24^. Several aspects need to be considered in order to evaluate the most suitable frequency for estimating abundance. First, the effect of resonance is a major problem affecting the lower frequencies (below 38 kHz) because small variations in size and depth lead to great differences in the *TS* values. Second, the low depth of penetration of the higher frequencies limits the maximum depth of study for biomass estimation. Finally, the acoustic contribution from other scatterers may have major effects at frequencies above 70 kHz. The use of the 120 kHz frequency has been recommended^33^ as it seems to be free from the resonance effect. The effect that slight changes in size and depth have on *TS* are less noticeable other than near or at the resonance frequency. This is valid even if it is subject to tilt angle variation. However, using frequencies ≥ 120 kHz is inappropriate^43^, especially for mesopelagic species, due to the noise derived from the low penetration depth associated with higher frequencies. In agreement with previous studies, the choice of the 38 kHz over 18 and 70 kHz is a compromise between reducing the effect of resonance, maximising depth of penetration and minimising the acoustic contribution from zooplankton^43^.

## Conclusions

In this work, we present acoustic measurements and dedicated pelagic trawls suitable to estimate biomass of pearlside. Vertical and horizontal distribution of pearlside as well as the daily migration patterns were obtained based on the acoustic measurements. We obtained measured *TS* and frequency-dependent dB differences at five different frequencies, including at 70 kHz, not published before for this species. The obtained results show a general decreasing response with frequency, consistent with a resonance below 38 kHz. In addition, we present for the first time *TS*-length relationships (*b*_20_) (−65.9 ± 2, −69.2 ± 3, −69.2 ± 2, −69.5 ± 2.5 and −71.5 ± 2.5 dB at 18, 38, 70, 120 and 200 kHz, respectively). An extensive set of morphological measures was obtained describing the general shape patterns of the swimbladder of this species for a wide range of fish lengths. A positive correlation was found between swimbladder size and body length, in agreement with the increasing *TS*-length relationship observed. The best agreement was obtained using a model that allowed full contraction of the swimbladder according to Boyle’s law, thus showing that, during the trawls, pearlside does not compensate swimbladder volume. Consequently, the actual equivalent sphere radius of pearlside at depth should be about 50% smaller than observed at the surface for the range of depths found in this study. The set of results reported in this study are essential for future pearlside biomass estimations and theoretical simulations, and key to evaluating the impact of their exploitation and establishing the necessary management measures.

## Supplementary information

Supplementary table 1

## Data Availability

Datasets generated and/or analysed during this study are available from the corresponding author upon reasonable request.

## References

[1] Williams A, Koslow J, Terauds A, Haskard K (2001). Feeding ecology of five fishes from the mid-slope micronekton community off southern Tasmania, Australia. Mar. Biol..

[2] Cherel Y, Ducatez S, Fontaine C, Richard P, Guinet C (2008). Stable isotopes reveal the trophic position and mesopelagic fish diet of female southern elephant seals breeding on the Kerguelen Islands. Mar. Ecol. Prog. Ser..

[3] Gjøsaeter, J. & Kawaguchi, K. *A review of the world resources of mesopelagic fish*. (Food & Agriculture Org., 1980).

[4] Sassa C, Kawaguchi K, Kinoshita T, Watanabe C (2002). Assemblages of vertical migratory mesopelagic fish in the transitional region of the western North Pacific. Fish. Oceanogr..

[5] Irigoien, X. *et al*. Large mesopelagic fishes biomass and trophic efficiency in the open ocean. *Nat. Commun*. **5**, (2014). 10.1038/ncomms4271 PMC3926006 24509953

[6] Prosch, R.M, Hulley, P.A. & Cruickshank, R.A. Mesopelagic fish and some other forage species. In *Oceans of Life off Southern Africa* 130–135 (1989).

[7] O’Driscoll RL, Gauthier S, Devine JA (2009). Acoustic estimates of mesopelagic fish: as clear as day and night?. ICES J. Mar. Sci..

[8] Savinykh VF, Baytalyuk AA (2010). New data on biology of pearlfish Maurolicus imperatorius (Sternopthychidae) from the Emperor Seamount Chain. J. Ichthyol..

[9] Prellezo R (2018). Exploring the economic viability of a mesopelagic fishery in the Bay of Biscay. ICES J. Mar. Sci..

[10] Springmann M (2018). Options for keeping the food system within environmental limits. Nature.

[11] Hidalgo, M. & Browman, H. I. Contribution to the Themed Section: ‘Mesopelagic resources’. (2019).

[12] Directorate-General for Maritime Affairs and Fisheries. Blue bioeconomy. Situation, report and perspectives. (2018).

[13] Godø OR, Patel R, Pedersen G (2009). Diel migration and swimbladder resonance of small fish: some implications for analyses of multifrequency echo data. ICES J. Mar. Sci..

[14] Proud R, Cox MJ, Brierley AS (2017). Biogeography of the Global Ocean’s Mesopelagic Zone. Curr. Biol..

[15] Proud Roland, Handegard Nils Olav, Kloser Rudy J, Cox Martin J, Brierley Andrew S (2018). From siphonophores to deep scattering layers: uncertainty ranges for the estimation of global mesopelagic fish biomass. ICES Journal of Marine Science.

[16] Jennings S, Collingridge K (2015). Predicting Consumer Biomass, Size-Structure, Production, Catch Potential, Responses to Fishing and Associated Uncertainties in the World’s Marine Ecosystems. PLOS ONE.

[17] Anderson TR (2018). Quantifying carbon fluxes from primary production to mesopelagic fish using a simple food web model. ICES J. Mar. Sci..

[18] Kaartvedt S, Staby A, Aksnes D (2012). Efficient trawl avoidance by mesopelagic fishes causes large underestimation of their biomass. Mar. Ecol. Prog. Ser..

[19] Peña Marian (2018). Mesopelagic fish avoidance from the vessel dynamic positioning system. ICES Journal of Marine Science.

[20] Koslow, J. A., Kloser, R. J. & Williams, A. Pelagic biomass and community structure over the mid-continental slope off southeastern Australia based upon acoustic and midwater trawl sampling. *Mar. Ecol. Prog. Ser*. 21–35 (1997).

[21] Kloser RJ, Ryan TE, Young JW, Lewis ME (2009). Acoustic observations of micronekton fish on the scale of an ocean basin: potential and challenges. ICES J. Mar. Sci..

[22] Pakhomov, E., Yamamura, O., Advisory Panel on Micronekton Sampling Inter-calibration Experiment & North Pacific Marine Science Organization. Report of the Advisory Panel on Micronekton Sampling Inter-calibration Experiment. (North Pacific Marine Science Organization (PICES), 2010).

[23] ICES. *Working Group for the Bay of Biscay and the Iberian waters Ecoregion (WGBIE) 7–13 May* 2*014 Lisbon, Portugal*. 748 (2014).

[24] Simmonds, E. J. & MacLennan, D. N. *Fisheries acoustics: theory and practice*. (Blackwell Science, 2005).

[25] Murase H (2009). Acoustic characterization of biological backscatterings in the Kuroshio-Oyashio inter-frontal zone and subarctic waters of the western North Pacific in spring. Fish. Oceanogr..

[26] Lezama-Ochoa A (2011). Spatial patterns and scale-dependent relationships between macrozooplankton and fish in the Bay of Biscay: an acoustic study. Mar. Ecol. Prog. Ser..

[27] Gastauer S, Scoulding B, Parsons M (2017). Estimates of variability of goldband snapper target strength and biomass in three fishing regions within the Northern Demersal Scalefish Fishery (Western Australia). Fish. Res..

[28] Madureira, L., Ward, P. & Atkinson, A. Differences in backscattering strength determined at 120 and 38 kHz for three species of Antarctic macroplankton. *Mar. Ecol. Prog. Ser*. 17–24 (1993).

[29] Kang M (2002). Effective and accurate use of difference in mean volume backscattering strength to identify fish and plankton. ICES J. Mar. Sci..

[30] Peña M (2014). Acoustic detection of mesopelagic fishes in scattering layers of the Balearic Sea (western Mediterranean). Can. J. Fish. Aquat. Sci..

[31] Torgersen T, Kaartvedt S (2001). *In situ* swimming behaviour of individual mesopelagic fish studied by split-beam echo target tracking. ICES J. Mar. Sci..

[32] Benoit-Bird KJ, Au WWL (2001). Target strength measurements of Hawaiian mesopelagic boundary community animals. J. Acoust. Soc. Am..

[33] Fujino T (2009). Swimbladder morphology and target strength of a mesopelagic fish, Maurolicus japonicus. J. Mar. Acoust. Soc. Jpn..

[34] Sawada K (2011). *In situ* and *ex situ* target strength measurement of mesopelagic lanternfish, Diaphus Theta (Family Myctophidae). J. Mar. Sci. Technol..

[35] Scoulding B, Chu D, Ona E, Fernandes PG (2015). Target strengths of two abundant mesopelagic fish species. J. Acoust. Soc. Am..

[36] Kloser RJ, Ryan T, Sakov P, Williams A, Koslow JA (2002). Species identification in deep water using multiple acoustic frequencies. Can. J. Fish. Aquat. Sci..

[37] Hamano A (1993). Studies on the acoustic method for estimating biomass of micronectonic fish. J. Shimonoseki Univ. Fish..

[38] Rasmussen OI, Giske J (1994). Life-history parameters and vertical distribution of Maurolicus muelleri in Masfjorden in summer. Mar. Biol..

[39] Foote KG (1980). Importance of the swimbladder in acoustic scattering by fish: A comparison of Gadoid and mackerel target strengths. J. Acoust. Soc. Am..

[40] Haslett RWG (1962). Determination of the acoustic backscattering patterns and cross sections of fish. Br. J. Appl. Phys..

[41] Blaxter JHS, Batty RS (1990). Swimbladder ‘behaviour’ and target strength. Rapp. Proces-Verbaux La Réun. Cons. Int. Pour L’Exploration Mer.

[42] Horne JK (2000). Acoustic approaches to remote species identification: a review. Fish. Oceanogr..

[43] Davison PC, Koslow JA, Kloser RJ (2015). Acoustic biomass estimation of mesopelagic fish: backscattering from individuals, populations, and communities. ICES J. Mar. Sci..

[44] Peña M, Calise L (2016). Use of SDWBA predictions for acoustic volume backscattering and the Self-Organizing Map to discern frequencies identifying Meganyctiphanes norvegica from mesopelagic fish species. Deep Sea Res. Part Oceanogr. Res. Pap..

[45] Kloser RJ, Ryan TE, Keith G, Gershwin L (2016). Deep-scattering layer, gas-bladder density, and size estimates using a two-frequency acoustic and optical probe. ICES J. Mar. Sci. J. Cons..

[46] Boyra G (2013). Acoustic surveys for juvenile anchovy in the Bay of Biscay: abundance estimate as an indicator of the next year’s recruitment and spatial distribution patterns. ICES J. Mar. Sci..

[47] Demer, D. A. *et al*. *Calibration of acoustic instruments* (2015).

[48] Appendix A of the European Convention for the protection of vertebrate animals used for experimental and other scientific purposes (ETS No. 123). Available at: https://rm.coe.int/CoERMPublicCommonSearchServices/DisplayDCTMContent?documentId=090000168007a445. (Accessed: 20th May 2019)

[49] *Echoview Software*. (Echoview Software Pty Lt, 2013).

[50] De Robertis A, Higginbottom I (2007). A post-processing technique to estimate the signal-to-noise ratio and remove echosounder background noise. ICES J. Mar. Sci. J. Cons..

[51] *R: A language and environment for statistical computing*. (R Core Team, 2017).

[52] Soule M (1997). Performance of a new phase algorithm for discriminating between single and overlapping echoes in a split-beam echosounder. ICES J. Mar. Sci..

[53] Peña H (2008). *In situ* target-strength measurements of Chilean jack mackerel (Trachurus symmetricus murphyi) collected with a scientific echosounder installed on a fishing vessel. ICES J. Mar. Sci. J. Cons..

[54] Gauthier S, Rose GA (2001). Diagnostic tools for unbiased *in situ* target strength estimation. Can. J. Fish. Aquat. Sci.

[55] Ona, E. & Barange, M. *Single target recognition*. 28–43 (1999).

[56] Barange M, Hampton I, Soule M (1996). Empirical determination of *in situ* target strengths of three loosely aggregated pelagic fish species. ICES J. Mar. Sci. J. Cons..

[57] MacLennan DN, Menz A (1996). Interpretation of *in situ* target-strength data. ICES J. Mar. Sci. J. Cons..

[58] Andreeva IB (1964). Scattering of sound by air bladders of fish in deep sound-scattering ocean layers..

[59] Weston, D. E. Sound propagation in the presence of bladder fish. In *Underwater Acoustics* 55–58 (Plenum, 1966).

[60] Love RH (1978). Resonant acoustic scattering by swimbladder-bearing fish ^a)^. J. Acoust. Soc. Am..

[61] Furusawa Masahiko (1988). Prolate spheroidal models for predicting general trends of fish target strength. Journal of the Acoustical Society of Japan (E).

[62] Ye Z (1997). Low-frequency acoustic scattering by gas-filled prolate spheroids in liquids. J. Acoust. Soc. Am..

[63] Strasberg M (1953). The pulsation frequency of nonspherical gas bubbles in liquids. J. Acoust. Soc. Am..

[64] Marshall N. B. *Swimbladder Structure of Deep-Sea Fishes in Relation to Their Systematics and Biology*. 27, (University Press, 1960).

[65] Ona E (2003). An expanded target-strength relationship for herring. ICES J. Mar. Sci..

[66] Peña M., González-Quirós R., Munuera-Fernández I., González F., Romero-Romero S., Nogueira E. (2019). Vertical distribution and aggregation patterns of krill (Crustacea: Euphausiacea) in the Bay of Biscay: interannual and seasonal variability. Canadian Journal of Zoology.

[67] Boyra G (2018). Target strength of skipjack tuna (Katsuwanus pelamis) associated with fish aggregating devices (FADs). ICES J. Mar. Sci..

[68] Demer DA, Soule MA, Hewitt RP (1999). A multiple-frequency method for potentially improving the accuracy and precision of *in situ* target strength measurements. J. Acoust. Soc. Am..

[69] Conti S, Demer D, Soule M, Conti J (2005). An improved multiple-frequency method for measuring target strengths. ICES J. Mar. Sci..

[70] Sawada K, Furusawa M, Williamson NJ (1993). Conditions for the precise measurement of fish target strength *in situ*. J. Mar. Acoust. Soc. Jpn..

[71] Korneliussen RJ, Ona E (2003). Synthetic echograms generated from the relative frequency response. ICES J. Mar. Sci..

[72] Yoon G-D, Shin H-H, Hwang K-S (1999). Target strength of fishes for estimating biomass - Distribution characteristics and target strength measurement of micronektonic fish, Maurolicus muelleri in the East Sea. Bull. Korean Soc. Fish. Tech..

[73] Nichol DG, Chilton EA (2006). Recuperation and behaviour of Pacific cod after barotrauma. ICES J. Mar. Sci..

[74] Love RH, Fisher RA, Wilson MA, Nero RW (2004). Unusual swimbladder behavior of fish in the Cariaco Trench. Deep Sea Res. Part Oceanogr. Res. Pap..

[75] Bali o BM, Aksnes DL (1993). Winter distribution and migration of the sound scattering layers, zooplankton and micronekton in Masfjorden, western Norway. Mar. Ecol.-Prog. Ser..

[76] Staby A, Srisomwong J, Rosland R (2013). Variation in DVM behaviour of juvenile and adult pearlside (*Maurolicus muelleri*) linked to feeding strategies and related predation risk. Fish. Oceanogr..

[77] Eggers DM (1978). Limnetic feeding behavior of juvenile sockeye salmon in Lake Washington and predator avoidance 1. Limnol. Oceanogr..

[78] Hrabik TR, Jensen OP, Martell SJD, Walters CJ, Kitchell JF (2006). Diel vertical migration in the Lake Superior pelagic community. I. Changes in vertical migration of coregonids in response to varying predation risk. Can. J. Fish. Aquat. Sci..

[79] Wurtsbaugh WA, Neverman D (1988). Post-feeding thermotaxis and daily vertical migration in a larval fish. Nature.

[80] Neilson, J. D. & Perry, R. I. Diel Vertical Migrations of Marine Fishes: an Obligate or Facultative Process? In *Advances in Marine Biology* (eds Blaxter, J. H. S. & Southward, A. J.) 26, 115–168 (Academic Press, 1990).

[81] Misund OA, Beltestad AK (1996). Target-strength estimates of schooling herring and mackerel using the comparison method. ICES J. Mar. Sci..

[82] Heino M (2011). Catchability of pelagic trawls for sampling deep-living nekton in the mid-North Atlantic. ICES J. Mar. Sci..

[83] Gartner J. JV (1988). Escapement by fishes from midwater trawls:a case study using lanternfishes (Pisces:Myctophidae). Fish.Bull..

[84] Itaya K, Fujimori Y, Shimizu S, Komatsu T, Miura T (2007). Effect of towing speed and net mouth size on catch efficiency in framed midwater trawls. Fish. Sci..

[85] Davison P, Lara-Lopez A, Anthony Koslow J (2015). Mesopelagic fish biomass in the southern California current ecosystem. Deep Sea Res. Part II Top. Stud. Oceanogr..

