# Supplementary material for: Target Strength and swimbladder morphology of Mueller’s pearlside (Maurolicus muelleri)
Source: Sci Rep. 2019 Nov 21;9:17311. doi: 10.1038/s41598-019-53819-6 (PMC6872731; doi:10.1038/s41598-019-53819-6)
Supplement: Supplementary file 1 — Supplementary table 1 [file 41598_2019_53819_MOESM1_ESM.pdf]

Target Strength and swimbladder morphology of Mueller´s pearlside (*Maurolicus muelleri)*

Sobradillo, B.[a]*, Boyra, G.[a], Martinez, U[a]., Carrera, P.[b], Peña, M.[c] and Irigoien, X.[a]

[a]Azti-Tecnalia. Herrera kaia, portualdea z/g, 20110, Pasaia, Spain.

[b]Instituto Español de Oceanografía, Vigo, Spain

[c]Instituto Español de Oceanografía, Centro Oceanográfico de Baleares, Palma de Mallorca, Spain.

E-mail addresses: bsobradillo@azti.es, gboyra@azti.es, umartinez@azti.es, pablo.carrera@ieo.es, marian.pena@ieo.es, xirigoien@azti.es

* Corresponding author. Telephone: +34 652.714.457

Supplementary information

Supplementary table 1. Model parameters.

Model parameters	Symbols	Units	Values
Sound speed	c_w	m s^{-1}	1490
Density of sea water	ρ_w	Kg m^{-3}	1026
Density of fish flesh	ρ_f	Kg m^{-3}	1050
Density of air	ρ_a	Kg m^{-3}	1.3
Ratio of specific heat for air (swimbladders)	γ_a	-	1.4
Specific heat at constant pressure for air (swimbladders)	cp_a	Cal kg^{-1} °C^{-1}	240
Surface tensión	s	N m^{-1}	200
Thermal conductivity of air	κ_a	Cal m^{-1} s^{-1} °C^{-1}	5.5 x 10^{-3}
Real part of complex shear modulus of fish tissue	μ_r	N m^{-2}	1 x 10^{6}
Complex part of shear modulus of fish tissue	μ_i	N m^{-2}	3 x 10^{4}
